# Early prediction of ventricular peritoneal shunt dependency in aneurysmal subarachnoid haemorrhage patients by recurrent neural network-based machine learning using routine intensive care unit data

**DOI:** 10.1007/s10877-024-01151-4

**Published:** 2024-03-21

**Authors:** Nils Schweingruber, Jan Bremer, Anton Wiehe, Marius Marc-Daniel Mader, Christina Mayer, Marcel Seungsu Woo, Stefan Kluge, Jörn Grensemann, Fanny Quandt, Jens Gempt, Marlene Fischer, Götz Thomalla, Christian Gerloff, Jennifer Sauvigny, Patrick Czorlich

**Affiliations:** 1 Department of Neurology, University Medical Center Hamburg-Eppendorf, 20246 Hamburg, Germany; 2 Department of Informatics, University of Hamburg, 22527 Hamburg, Germany; 3 Department of Neurosurgery, University Medical Center Hamburg-Eppendorf, Martinistr. 52, 20246 Hamburg, Germany; 4 Institute for Stem Cell Biology and Regenerative Medicine, Stanford University School of Medicine, Stanford, CA 94305 USA; 5 Institute of Neuroimmunology and Multiple Sclerosis (INIMS), Center for Molecular Neurobiology Hamburg (ZMNH), University Medical Center Hamburg-Eppendorf, 20246 Hamburg, Germany; 6 Department of Intensive Care Medicine, University Medical Center Hamburg-Eppendorf, 20246 Hamburg, Germany

**Keywords:** Machine learning, Neural networks (computer), Subarachnoid haemorrhage, Intensive care unit, Ventricular peritoneal shunt, Hydrocephalus

## Abstract

Aneurysmal subarachnoid haemorrhage (aSAH) can lead to complications such as acute hydrocephalic congestion. Treatment of this acute condition often includes establishing an external ventricular drainage (EVD). However, chronic hydrocephalus develops in some patients, who then require placement of a permanent ventriculoperitoneal (VP) shunt. The aim of this study was to employ recurrent neural network (RNN)-based machine learning techniques to identify patients who require VP shunt placement at an early stage. This retrospective single-centre study included all patients who were diagnosed with aSAH and treated in the intensive care unit (ICU) between November 2010 and May 2020 (*n* = 602). More than 120 parameters were analysed, including routine neurocritical care data, vital signs and blood gas analyses. Various machine learning techniques, including RNNs and gradient boosting machines, were evaluated for their ability to predict VP shunt dependency. VP-shunt dependency could be predicted using an RNN after just one day of ICU stay, with an AUC-ROC of 0.77 (CI: 0.75–0.79). The accuracy of the prediction improved after four days of observation (Day 4: AUC-ROC 0.81, CI: 0.79–0.84). At that point, the accuracy of the prediction was 76% (CI: 75.98–83.09%), with a sensitivity of 85% (CI: 83–88%) and a specificity of 74% (CI: 71–78%). RNN-based machine learning has the potential to predict VP shunt dependency on Day 4 after ictus in aSAH patients using routine data collected in the ICU. The use of machine learning may allow early identification of patients with specific therapeutic needs and accelerate the execution of required procedures.

**Supplementary Information:**

The online version contains supplementary material available at 10.1007/s10877-024-01151-4.

## Introduction

Patients with aneurysmal subarachnoid haemorrhage (aSAH) receive interdisciplinary treatment in the intensive care unit (ICU) after the undergo primary acute interventions. Often, secondary complications, such as acute hydrocephalic congestion, vasospasms, or delayed cerebral ischaemia (DCI), occur [1–3]. In particular, secondary acute hydrocephalic congestion is life-threatening and is usually treated with the establishment of an external ventricular drainage (EVD). Cerebrospinal fluid (CSF) drainage is often necessary, but it is only temporarily. The process of gradual EVD weaning begins when patients meet certain criteria related to the resolution of hydrocephalus, such as changes in the quantity and quality of CSF output, intracranial pressure (ICP), and neurological stability [4]. Early EVD weaning is less strongly associated with ventriculoperitoneal (VP) shunt dependency [5]. A meta-analysis revealed that risk factors for shunt dependency included a high Fisher grade, the presence of acute hydrocephalus, in-hospital complications, the presence of intraventricular blood, a high Hunt and Hess scale score, rebleeding, a posterior circulation location of the aneurysm, and age ≥ 60 years [4].

Deep learning methods can improve the treatment of severely ill patients by enabling experienced caregivers to make objective decisions, with these decisions being made via a broader spectrum of caregivers, and the ability of deep learning methods to help make decisions has been demonstrated in sepsis therapy [6] and the prediction of circulatory [7] or renal failure [8]. In the ICU, recurrent neural networks (RNNs) with routine ICU data were used to predict critical ICP phases [9].

A machine learning prognostic model using a distributed random forest algorithm that includes 21 variables, such as radiological information and the occurrence of vasospasm, was found to accurately predict shunt dependency in patients with aSAH [10]. Another study suggested that the performance of functional outcome prediction by machine learning techniques was comparable to that of traditional methods and established clinical scores, with no significant difference in performance between traditional and other machine learning (ML) applications, and the most important variables were GCS score and age [11]. The XGBoost algorithm (extreme gradient boosting machine) has been shown to be more accurate than a logistic regression model in predicting outcomes for patients with aSAH and is helpful in identifying high-risk aSAH patients for improved medical care [12].

Overall, at the beginning of ICU treatment, it remains difficult to predict which patients will develop a VP-shunt dependency. Numerous studies on this topic have focused on primary aSAH-specific data, such as blood volume and blood distribution, the Graeb score, the presence of acute hydrocephalus and CSF dynamics within the first days after ictus [13, 14].

Objective clinical routine ICU data are easy to retrieve automatically, and the identification of patients at high risk can be facilitated if clinical routine ICU data during the clinical workflow are combined with machine learning techniques. The easy accessibility of medical data due to digital storage in combination with recent developments in the field of ML has the potential for automatized data processing to make predictions, look for certain patterns, and classify these data.

The aim of this study was to examine the potential of routine ICU data collected using RNN-based machine learning techniques to predict the development of chronic shunt-dependent hydrocephalus at an early stage.

## Methods

### Study design, setting and ethics

All patients who presented to the ICU with aSAH between 10/2010 and 05/2020 were included in this retrospective cohort study. Our institutional and interdisciplinary Department of Intensive Care Medicine operates 140 high-care ICU beds and treats approximately 5,700 patients per year. The study protocol was reported to the local ethics committee (Ethics Committee of the Hamburg Chamber of Physicians, reference number WF-059/20) and was conducted according to the Declaration of Helsinki. Written informed consent was waived because all datasets of the study were deidentified prior to processing and evaluation. The aneurysmal nature of the haemorrhage of each patient was verified by cerebral digital subtraction angiography, head-CT angiography (CTA), and/or magnetic resonance imaging (MRI) angiography. Patients in whom no aneurysm could be identified or who died prior to sufficient diagnostics were included in a separate analysis described in Supplementary Tables 1 and Supplementary Fig. 1. The occurrence of vasospasm and DCI was defined according to the criteria published by Vergouwen et al. [15] In patients with proven high-grade vasospasm on CTA and/or a perfusion deficit on perfusion computed tomography (CTP), digital subtraction angiography (DSA) was then carried out, and subsequent intraarterial nimodipine treatment was given. Acute hydrocephalus was diagnosed by experienced senior physicians on the basis of image morphology in correlation with the clinical presentation of the patients. EVD-related ventriculitis was defined as reported by the Centers for Disease Control and Prevention. Additionally, in patients with impaired consciousness or neurological deficits ventriculitis was suspected, and the treatment indications were based solely on previously described pathological CSF parameters, such as increased leucocytes, elevated protein, decreased absolute glucose, or a decreased CSF/serum glucose ratio if no growth was detected in the CSF culture in these patients [16, 17]. At our institution, we performed standardized gradual weaning of the EVD with a continuous increase in the outflow resistance up to 30 cmH_2_O above the foramen of Monro. The EVD was then closed for 48 h with parallel continuous measurement of the ICP. If there was no increase in the ICP, native noncontrast head CT (NCHCT) imaging was performed after 48 h, and the EVD was removed if hydrocephalus was ruled out by image morphology.

### Participants and data sources

The ICU is equipped with Dräger Delta vital sign monitoring systems (Drägerwerk, Lübeck, Germany). Patient information, laboratory values, blood gas analysis (BGA), and vital parameters were obtained from the electronic patient records (Integrated Care Manager V10, Drägerwerk, Lübeck, Germany) with its dedicated data retrieval software (ICMiq V1.4, Drägerwerk, Lübeck, Germany). Data collection also included demographic information, aSAH-specific information, such as aneurysm location, and distinct clinical evaluation scores such as the Glasgow Coma Scale (GCS), WFNS grading system, Hunt & Hess score, and Fisher score. The extent of intraventricular haemorrhage was measured using the original Graeb score [18]. 

### Preprocessing

Preprocessing was carried out as previously described [9]. The deidentified data were processed using the R programming language and its Tidyverse package, [19] as well as Python (pandas, numpy). The complete preprocessing procedure, including the list of features, is described in Supplementary Tables 2 and can be accessed via the publicly available GitHub repository: https://github.com/agschweingruber/sah. Blood gas analysis (BGA) and laboratory results are stored automatically in the system and are updated in real time. Some laboratory values, such as C-reactive protein (CRP) and white blood cell count, were obtained once daily, while a BGA was performed at least every four hours for patients undergoing invasive ventilation. All physicians and nursing staff were trained in the documentation system and digitally documented vital signs at least hourly or in response to special events. The medication and its dosage were manually assigned in the system and were not changed automatically. Scores were entered using drop-down menus in the software interface, and a dictionary of defined groups based on string values was also used to support the study. We implemented data standardization by averaging the recorded values on an hourly basis due to their nonstandard original intervals. This affected mainly the vital parameters, which were recorded at a resolution varying between 30 and 60 min, as determined by caregivers or physicians.

### Supervised learning

Three machine learning models were employed and compared: extreme gradient boosting (XGBoost [20]), long short-term memory (LSTM [21]) and logistic regression. The LSTM model is a type of RNN that can process sequences of data and determine whether the information is retained or discarded. The initial seven days of data were utilized as the primary input for the analysis. Clinical scores at baseline for patients with aSAH were used as a baseline for predicting the dependence on a VP shunt. We aimed to develop a model that is robust to raw datasets with missing features from various clinical sources. Model training and tuning were conducted using a nested K-fold cross-validation approach. This approach involves dividing the data into six parts and using one part as the held-out test set while training the model on the remaining five parts. Division was stratified according to the target. This process was repeated six times to obtain a more robust estimate of model performance.

The RNN model class employs an effective combination of multilayer perceptron (MLP) and LSTM networks. A bidirectional LSTM, which was chosen for its ability to understand patterns in sequential data, is central to its design. The model starts with an input dimension of 149—the number of input features. The initial processing of the data is performed through an MLP. This involves a series of linear layers and rectified linear unit (ReLU) activations that streamline the input into a more manageable form, reducing it to a dense dimension of 32. These refined data are then fed into the LSTM layer. LSTM, set up with a bidirectional structure and a 32-dimensional layer, is adept at analysing the temporal aspects of the data, considering both past and future contexts. The LSTM’s output is further processed through linear layers and ReLU activations, resulting in the final classification into two classes (No VP-Shunt vs. VP-Shunt).

### Statistics

The performances of XGBoost, RNN and logistic regression algorithms were compared against the abovementioned traditional aSAH scores. To obtain the receiver operating characteristic (ROC) curve, the sensitivity (recall), specificity, and precision were calculated. Predictions of deep learning models are continuous, and a threshold must be set to classify a prediction as true or false. To further display the performance of ML models independent of a set threshold, ROC and precision recall (PR) are used. ROC curves simulate the trade-off between specificity and sensitivity (a perfect classifier would have an area under the ROC curve (AUC-ROC) of one). To optimize the prediction thresholds, we conducted a thorough tuning process within each test-fold of the nested cross-validation, examining threshold values from 0 to 1 in steps of 0.01. The optimal threshold was determined by the highest F1-score for each individual test-fold. By applying these optimal thresholds to dichotomize predictions for each fold, we computed the sensitivity, specificity, F1-score, and accuracy. These metrics represent the mean values and confidence intervals calculated from each fold at the thresholds associated with the best F1-scores. The AUC was calculated using the sklearn package. The model was deployed in a 6-fold nested cross-validation scheme. The mean and the confidence intervals were calculated based on the prediction of six independent testing folds. The metrics were calculated from only the data of patients who were discharged alive. To compare the models and their corresponding scores, a bootstrapping technique was employed, generating 1000 resampled datasets from the differences in ROC-AUC scores. Subsequently, a one-sample t test was performed for each set of differences, and the results were compared against a null hypothesis value of 0. Notably, all bootstrapped differences satisfied the Kolmogorov‒Smirnov test with a significance level of *p* < 0.001, indicating their statistical validity. P values < 0.05 were considered significant. Visualization was performed using matplotlib. Tables were created using the gt table package and pandas. Baseline patient characteristics were compared using the aov R package. Post finishing (layout and alignment of text) was performed using Adobe Illustrator.

### Feature importance

Neural networks can have complex architectures (multiple layers) and certain randomness in classification (bias). In essence and practical terms, the feature importance of recurrent ML models indicates the potential role of an input feature for a certain prediction. In contrast to normal statistical models, neural networks can have a certain randomness of prediction, which is also true when they evaluate input features. In a feature importance method, the calculations are repeated several times to determine the average importance of each feature. To calculate feature importance, SHAP [22] values were calculated.

### Code Availability

The code is available at https://github.com/agschweingruber/sah.

## Results

### Characteristics of patients receiving a VP shunt

**Fig. 1:**
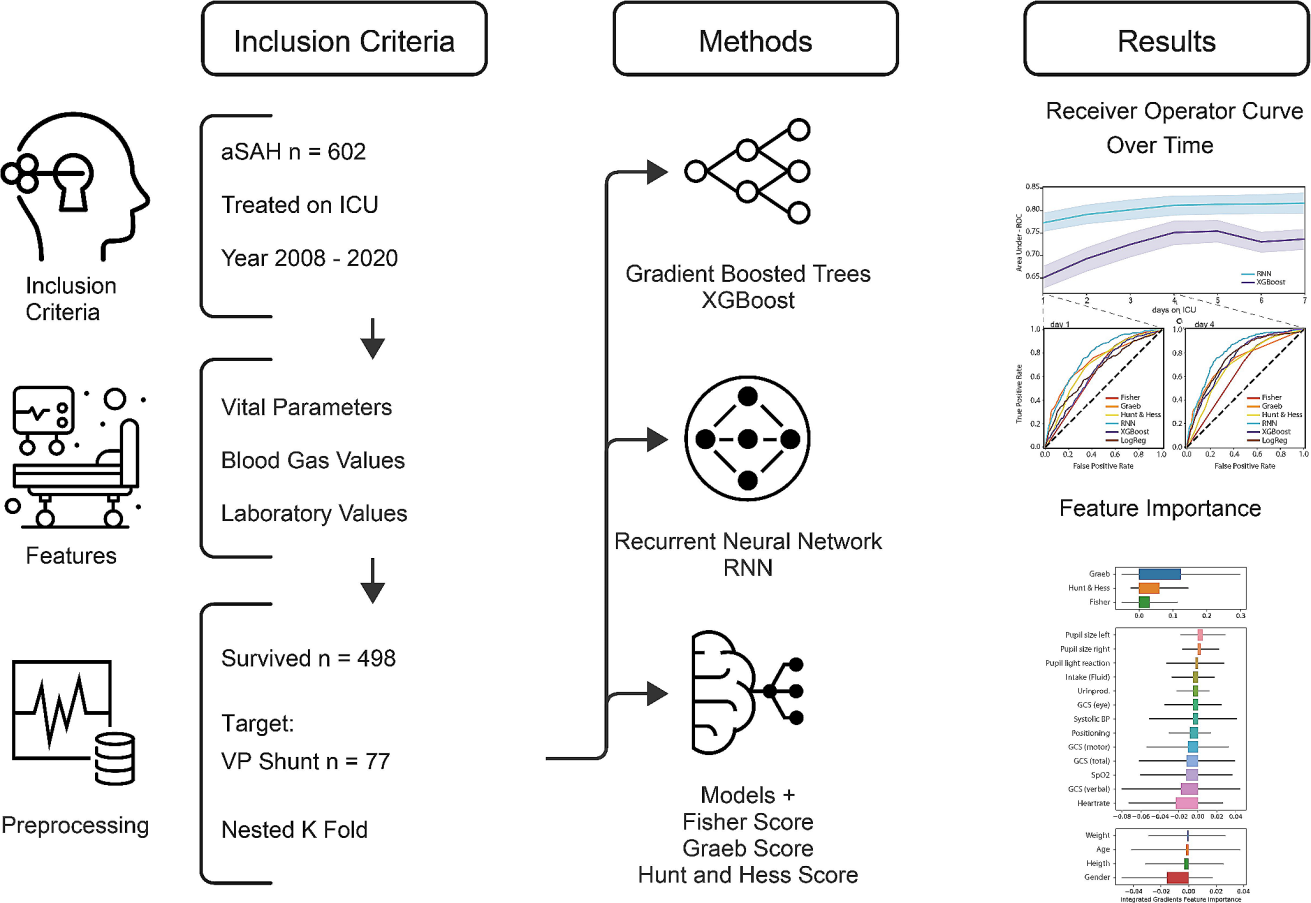
Visual abstract. Graphical representation of the main findings and key information of the present study. The inclusion criteria, methods used, and main results of the study are shown. The goal of this visual abstract is to provide a quick and easy-to-understand overview of the study

Of the 602 patients with aSAH included in the study, 77 (12.8%) required a VP shunt due to chronic hydrocephalus after weaning from EVD failure (Fig. 1; Table 1). The ICU treatment phase survived for all patients in the VP shunt group and for 421 patients (80.2%) in the Non-VP shunt group. Patients receiving a VP shunt had significantly greater scores on the Hunt and Hess scale, the WFNS test, the Fisher test and the original Graeb test. The most pronounced differences were observed in the Graeb score because the VP-shunt group had a median Graeb score of 5 (IQR 6) and the non-VP shunt groups had a median score of 2 (IQR 5) (*p* < 0.0001). The initial GCS score did not significantly differ between the two groups. The location of the aneurysm and the initial treatment procedure (endovascular or microsurgical) were not significantly different, although slightly more patients with aneurysms located in the posterior circulation received a VP shunt (40.3% vs. 30.7%, *p* = 0.092).

**Table 1:** Patient characteristics

	Non-VP-Shunt group	VP- Shunt Group	*p*-value
n	525 (100.0%)	77 (100.0%)	0.702
Male	177 (33.7%)	18 (23.3%)	0.070
Age (year)	55.2 (+-13.3)	57.7 (+-13.9)	0.131
Aneurysm anterior circ.	364 (69.3%)	46 (59.7%)	0.092
Aneurysm diameter (mm)	7.08 (+-5.13)	7.06 (+-4.46)	0.973
EVD-related ventriculitis	129 (24.6%)	46 (59.7%)	< 0.0001
Rebleeding	61 (11.6%)	21 (27.3%)	< 0.0001
Intraventricular blood	256 (52.4%)	57 (80.3%)	< 0.0001
Pupils normal on admission	455 (86.7%)	65 (84.4%)	0.591
Initial ICP (mmHg)	7.16 (+-13.9)	5.99 (+-8.60)	0.487
Length of ICU stay (days)	14.9 (+-9.74)	33.0 (+-10.9)	< 0.0001
Treatment			
Microsurgical	153 (29.1%)	20 (26.0%)	0.538
Endovascular	337 (64.2%)	57 (74.0%)	0.129
None	35 (6.7%)	NA	NA
Scores			
Hunt and Hess	2.0 (+-2.0)	3.0 (+-2.0)	< 0.001
WFNS	1.0 (+-3.0)	3.0 (+-3.0)	0.022
GCS	15.0 (+-9.0)	13.0 (+-9.0)	0.123
Fisher	4.0 (+-1.0)	4.0 (+-0)	< 0.001
Graeb	2.0 (+-5.0)	5.0 (+-6.0)	< 0.0001
Line			
Lumbar drainage	62 (11.8%)	35 (45.5%)	< 0.0001
EVD	304 (57.9%)	69 (89.6%)	< 0.0001
Parenchymal ICP Probe	62 (11.8%)	20 (26.0%)	< 0.001
Outcome			
Deceased	104 (19.8%)	NA	NA

EVD = external ventricular drainage; GCS = Glasgow Coma Scale; ICP = intracranial pressure; ICU = Intensive Care Unit; WFNS = World Federation of Neurosurgical Societies

The prevalence of CSF drainage was significantly greater in patients who developed VP shunt dependency than in those in the non-VP shunt group (EVD: 89.5% vs. 57.9%, *p* < 0.0001; lumbar drain: 45.5% vs. 11.8%, *p* < 0.0001). Complications such as intraventricular blood (80.3% vs.52.4%, *p* < 0.0001), ventriculitis (59.7% vs. 24.6%, *p* < 0.0001), and rebleeding (27.3% vs. 11.6%, *p* < 0.0001) were more frequent in the VP shunt group. Additionally, DCI (53.2% vs. 36.2%, *p* = 0.004) and vasospasm (79.2% vs. 57.8%, *p* < 0.001) were also more prevalent in the VP shunt group.

### Comparison of the XGBoost and RNN algorithms for predicting VP shunt dependency in aSAH patients

**Fig. 2:**
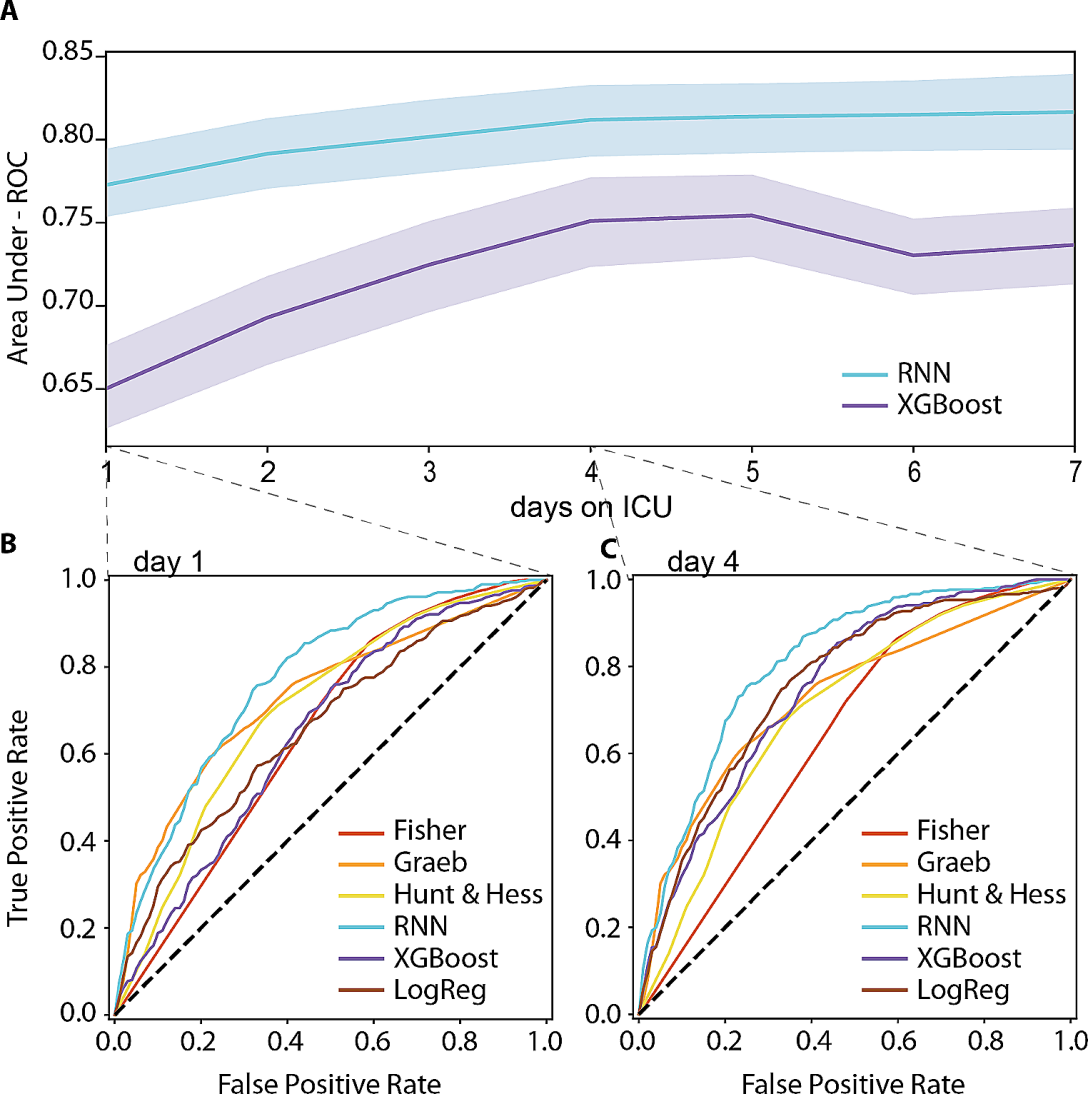
Prediction of VP shunt dependency: model performance over the first week with maximum accuracy on day 4. The figure represents the performance of two machine learning models, the recurrent neural network (RNN) and extreme gradient boosting (XGBoost) models, in predicting VP-Shunt dependency in aSAH patients admitted to the ICU. The figure is composed of three subplots (**A-C**). Subplot A shows the performance of the models over the first 7 days after ICU admission, with the RNN model represented by a blue line and the XGBoost model represented by a purple line. The lighter shaded area represents the confidence interval (CI) of the model performance. Subplot B presents the area under the curve (AUC) receiver operating characteristic (ROC) curve for the performance on Day 1, while subplot C presents the AUC-ROC plot for the performance on Day 4. The RNN and XGBoost models are represented by the same colours as in Subplot A, while the aSAH scores are depicted as the Fisher score in red, the Grab score in orange, the Hunt and Hess score in yellow and logistic regression in brown. It is worth noting that the CI is intentionally left out of subplots B and C to improve readability, as shown in Table 2

The Graeb score, a traditional aSAH score, demonstrated the greatest ability to discriminate between patients who received a VP shunt and those who did not (Table 2). The Graeb score had an area under the receiver operating characteristic curve (AUC-ROC) of 0.73 (CI: 0.69–0.77), a specificity of 0.78 (CI: 0.72–0.83), a sensitivity of 0.66 (CI: 0.58–0.74), and an accuracy of 0.76 (CI: 0.72–0.8). A comparison of the performances of the machine learning algorithms trained using time-dependent information from the ICU for the first days of the ICU stay is shown in Fig. 2. The results indicated that the RNN was superior to the XGBoost model as of Day 1 (RNN AUC-ROC: 0.77, CI 0.75–0.79; XGBoost AUC-ROC: 0.65, CI 0.62–0.68, *p* < 0.001), and its performance continued to improve over the first 4 days before stabilizing (Day 4: RNN AUC-ROC: 0.81, CI 0.79–0.83; XGBoost AUC-ROC: 0.75, CI 0.72–0.78, *p* < 0.001). A detailed confusion matrix can be found in Supplementary Fig. 2. The performance of XGBoost improved until Day 4 but then declined. The AUC-ROC of the best performing RNN was found to be superior to that of the Graeb score as of Day 1 (AUC-ROC RNN: 0.77, CI 0.75–0.79; AUC-ROC Graeb: 0.73, CI 0.69–0.77, *p* < 0.001) and was even more pronounced on Day 4, with the lower CI of the RNN (0.79) exceeding the upper CI of the Graeb score (0.77; AUC-ROC RNN Day 4: 0.82, CI 0.79–0.84; AUC-ROC Graeb: 0.73, CI 0.69–0.77, p = < 0.001)). The results for a temporal train-test split can be found in Supplementary Table 3.

**Table 2:** Model performance in survived aSAH patients treated on ICU to predict VP-shunt dependency

	**Day**	**Specificity**	**Sensitivity**	**Accuracy**	**AUC-ROC**	**F1**	**Average Precision**
RNN	1	0.72 (0.69–0.75)	0.78 (0.74–0.82)	0.73 (0.7–0.75)	0.77 (0.75–0.79)	0.37 (0.36–0.38)	0.42 (0.38–0.45)
	4	0.72 (0.69–0.76)	0.86 (0.83–0.89)	0.74 (0.71–0.77)	0.81 (0.79–0.83)	0.39 (0.38–0.4)	0.47 (0.43–0.51)
	7	0.74 (0.71–0.78)	0.85 (0.83–0.88)	0.76 (0.73–0.79)	0.82 (0.79–0.84)	0.39 (0.38–0.41)	0.48 (0.44–0.52)
XGBoost	1	0.61 (0.58–0.65)	0.71 (0.67–0.76)	0.63 (0.6–0.66)	0.65 (0.62–0.68)	0.32 (0.31–0.33)	0.28 (0.25–0.31)
	4	0.69 (0.66–0.72)	0.78 (0.75–0.81)	0.71 (0.68–0.73)	0.75 (0.72–0.78)	0.36 (0.35–0.37)	0.38 (0.34–0.43)
	7	0.68 (0.65–0.71)	0.77 (0.73–0.8)	0.7 (0.67–0.72)	0.74 (0.71–0.76)	0.36 (0.35–0.37)	0.32 (0.29–0.35)
LogReg	1	0.66 (0.61–0.7)	0.67 (0.62–0.73)	0.66 (0.62–0.7)	0.66 (0.62–0.7)	0.32 (0.31–0.34)	0.32 (0.29–0.36)
	4	0.7 (0.67–0.73)	0.8 (0.75–0.84)	0.71 (0.69–0.74)	0.76 (0.73–0.79)	0.37 (0.36–0.38)	0.39 (0.36–0.43)
	7	0.73 (0.7–0.76)	0.8 (0.77–0.83)	0.74 (0.71–0.76)	0.77 (0.74–0.79)	0.38 (0.37–0.39)	0.41 (0.38–0.44)
Fisher	1	0.44 (0.41–0.47)	0.83 (0.77–0.89)	0.5 (0.47–0.52)	0.65 (0.62–0.67)	0.28 (0.27–0.29)	0.21 (0.2–0.23)
Graeb	1	0.78 (0.72–0.83)	0.66 (0.58–0.74)	0.76 (0.72–0.8)	0.73 (0.69–0.77)	0.34 (0.31–0.36)	0.36 (0.32–0.4)
Hunt& Hess	1	0.67 (0.65–0.69)	0.68 (0.62–0.74)	0.67 (0.66–0.69)	0.7 (0.68–0.73)	0.33 (0.32–0.34)	0.26 (0.24–0.28)

AUC-ROC = area under the receiver operating characteristic curve; aSAH = aneruysmal subarachnoid hemorrhage; ICU = intensive care unit; LogReg = logistic regression; RNN = recurrent neural network; VP = ventriculoperitoneal; XGBoost = Extreme Gradient Boosting; Depicted values are the mean and the confidence intervals calculated based on the prediction of six independent testing folds

### Top features influencing VP-Shunt dependency prediction in the RNN Model

**Fig. 3:**
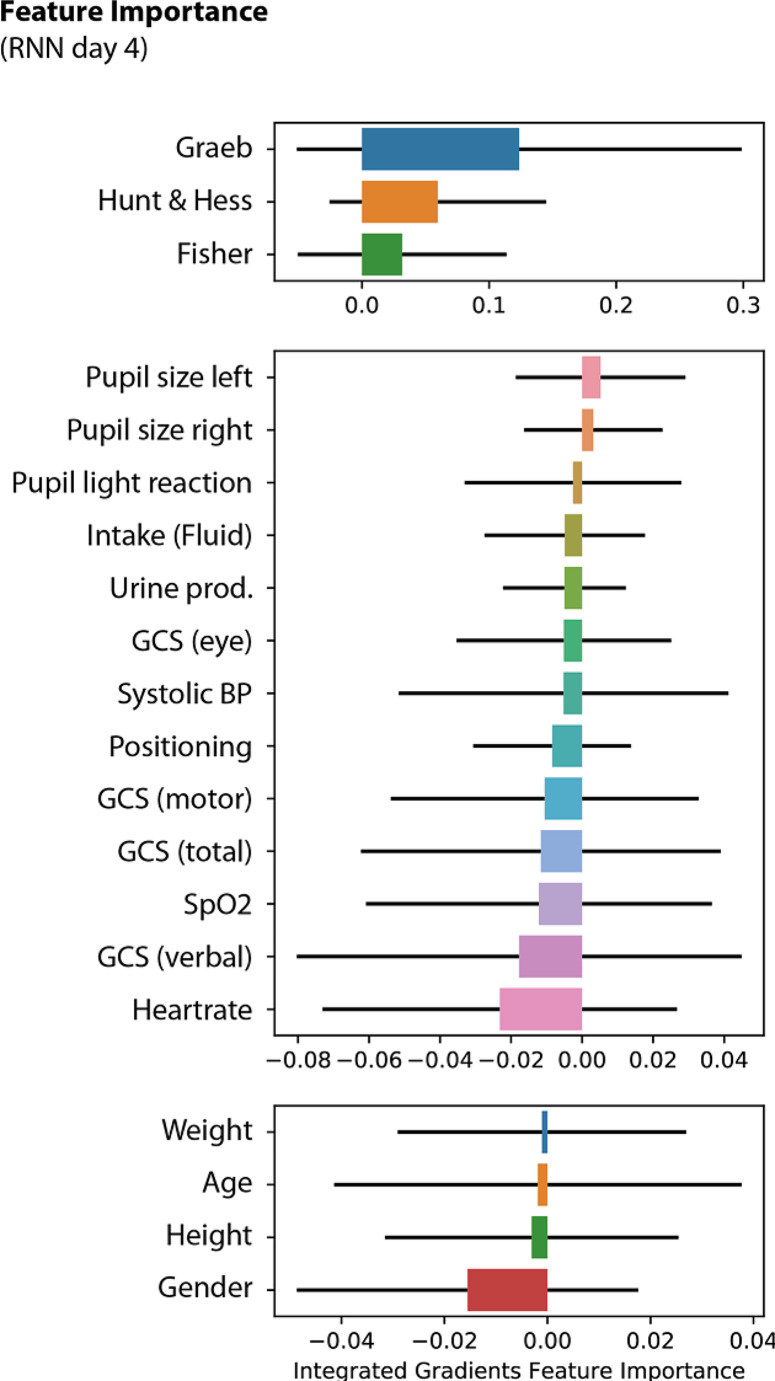
Feature importance of clinical data in RNN model prediction of VP-shunt dependency on day 4 of ICU admission. Feature Importance Plot on Day 4 of the Recurrent Neural Network Model. The plot represents the top 20 features that contribute most to the prediction of VP-shunt dependency, as calculated using the integrated gradients method. The standard deviation is depicted as a black line, and the features are divided into three groups based on their origin: aSAH scores (upper), vital parameters (middle), and baseline patient characteristics (lower). The names of the individual features are displayed in the figure. The standard deviation of the feature importance is depicted as a black line

The top 20 features of the best-performing RNN (Day 4) were analysed for feature importance. By analysing the importance of the features from our optimal RNN model (Fig. 3), we found notable correlations. Primarily, the Graeb score was the most influential feature. Pupil size had a positive correlation with VP shunt dependency. Conversely, the pupil light reaction had a bidirectional influence. Lower levels of fluid intake, urine production, and GCS (including subscores, particularly eye sub-scores) were associated with greater probabilities of VP shunt dependency. Patients who spent more time in bed, indicated by lower positioning values, also had a greater likelihood of VP shunt dependency. Vital parameters such as systolic blood pressure, blood oxygen saturation, and heart rate also showed a relationship, with lower values indicating a greater likelihood of VP shunting. Demographics such as age, weight, and height had less influence on the predictions, but female sex had a positive correlation with VP shunting.

## Discussion

Our study demonstrated that machine learning algorithms utilizing routine clinical data automatically retrieved from ICUs have the potential to predict VP shunt dependency in patients with aSAH. Moreover, using ML to evaluate scores and clinical data outperformed established prognostic and imaging scores with respect to the prediction of dependency on the VP shunt. In the comparison of the performance of the XGBoost and RNN algorithms for predicting VP shunt dependency in aSAH patients, the RNN algorithm was found to be superior to the XGBoost model. The top 20 features influencing VP-shunt dependency prediction in the RNN model were analysed and found to rely on imaging scores, particularly the original Graeb score, as well as vital signs such as heart rate and oxygen saturation.

The baseline characteristics of the VP-shunt group differed significantly from those of the non-VP-shunt group, with the main discrepancy being observed in bleeding scores. This reflects variations in both the amount and location of bleeding. In particular, scores such as the Graeb score, which quantifies intraventricular bleeding, have been shown to better discriminate VP shunt dependence than other predictors such as the Fisher scale [23]. Quantifying haemorrhage volume provides an independent predictor of hydrocephalus in patients with aSAH [24]. The implementation of machine learning algorithms on NCHCT scans of aSAH patients could improve accuracy over that of subjective scoring systems and reduce interrater variability. In this study, the Graeb score was evaluated, and it demonstrated the highest AUC-ROC for detecting VP shunt dependence among the other aSAH scores. The RNN was found to be the most accurate model for predicting VP shunt dependence as early as day one, although its performance was not significantly better than that of the scores, and the scores were used as input for the RNN. This finding is consistent with previous studies, which have shown no clear superiority in predicting shunt dependence using machine learning [11]. 

However, when additional information, such as the occurrence of vasospasms or infarction, is added to the algorithm, machine learning models have been shown to outperform other methods [12]. It is important to note that because these published systems provide some information about the end of treatment, they lack practical applicability in real-world settings [18]. Another limitation of some scores, such as the CHESS score, is the significantly poorer predictive value of external validations [11, 13]. This limitation is lower with machine learning models, as these models can be updated and refined over time with new data, enabling the continuous improvement in their predictive performance. Moreover, machine learning models can be easily integrated into routine clinical workflows and can automate the process of quantifying haemorrhage volumes, making them more practical than scores that require manual rating.

In their recent investigation, Frey et al. provided evidence supporting the efficacy of machine learning methodologies in enhancing the accuracy of established prognostic models for shunt-dependent hydrocephalus [25]. In this study, increased predictive ability was achieved through the incorporation of the 14-day CSF volume parameter. In contrast, our methodology yielded comparable predictive accuracy using data obtained as early as the fourth day postonset. The predictive efficacy of our approach is in line with the outcomes of various studies, even when divergent data analysis strategies are employed [18, 25]. Notably, studies incorporating variables recorded at the culmination of treatment, such as the duration of ICU stay, have demonstrated superior predictive performance [10]. 

The RNN proposed in this study, however, has the potential for real-world application because it can be run in the background of an ICU system and can continuously predict VP shunt dependence once all relevant information about the type of aSAH and baseline characteristics has been added. The predictions are expected to plateau on day four, providing sufficient time for the initiation of clinical interventions in order to manage complications in aSAH patients, as VP-shunt dependence is associated with numerous events during an ICU stay. Advanced knowledge of this could lead to more proactive monitoring and management of these patients. The impact of early classification of these patients on the management of aSAH patients requires further investigation through a prospective clinical trial, and this early classification can help identify high-risk aSAH patients for improved medical care. However, by providing an accurate early indication of shunt dependency, unnecessary interventions such as the premature removal of an EVD and subsequent need for alternative drainage could be avoided. On the other hand, the tool could also help identify patients not at risk of VP shunt dependency, allowing for earlier or accelerated EVD weaning. This could minimize the time that CSF drainage is needed, possibly reducing associated risks and enhancing patient comfort. Further advanced planning and evaluation of the procedure could reduce the length of stay in the ICU, thereby accelerating patient transfer towards specialized rehabilitative care, reducing the risk of ICU-associated comorbidities such as infections with multidrug-resistant organisms, and lowering overall health care costs [26]. 

The results of this study indicate that the performance of the XGBoost algorithm decreased after Day 4 and was less robust than that of the RNN. This can be attributed to the superior ability of the RNN architecture to detect long-term dependencies. While the XGBoost algorithm may perform similarly, it requires more effort in terms of feature engineering to incorporate time-dependent information.

The proposed feature importance method was implemented to capture both positive and negative influences, as it is important to understand duality when examining the feature importance of neural networks. These models can help to make context-dependent decisions, and the importance of input features can vary depending on their surroundings. Hence, the importance of individual features should always be considered when applying these systems in real-world settings. However, questions about potential associations between these features and treatment outcomes have been raised. It is important to note that these algorithms are not designed to make treatment decisions or influence outcomes. Nevertheless, they provide insights into the decisions made based on the underlying training data. In this study, the continuous documentation of the GCS had an impact on the predictions made. The ability to communicate verbally had a positive influence on reducing shunt dependency, which may be due to better monitoring of patients who can express what symptoms they are experiencing during EVD weaning. Interestingly, male sex was found to be associated with a reduced risk of VP shunt dependency, a finding that has not been previously reported in the literature. This finding may stem from patterns that coincide with male sex and result in a lower probability of VP shunt dependency. However, caution should be exercised when applying these results, as the effects of these results on improving patient management has not been conclusively shown. The findings from this study offer additional insights into sex-specific disparities in outcomes following aSAH, particularly with regard to the elevated incidence of DCI among female patients, as previously shown [27]. 

The limitations of this study are primarily due to its monocentric design and lack of external validation. Efforts were made to estimate the model performance on external data using the nested k-fold method, but external validation is necessary for real-world applicability. Another limitation is the analysis of only the surviving patient population, and the potential implications for patients who did not survive were not evaluated. A system that predicts patient outcomes, including death, could address this limitation in future research.

Additionally, the differences in baseline characteristics between patients can affect the early performance of the algorithm. The scores used in the study still require a human interpretation of the initial CT scans, and the development of a system capable of extracting this information directly from the scans is a potential future goal. The Graeb score remains a cost-effective metric for distinguishing shunt dependency, but its performance may vary between clinics.

## Conclusions

Our study demonstrated that machine learning algorithms, specifically deep learning techniques, utilizing routine clinical data automatically retrieved from the ICU have the potential to predict VP shunt dependency in patients with aSAH. With the evolution and advancement of data acquisition methods, we anticipate that these algorithms will improve in the future, and they can potentially outperform traditional scores. The implementation of such machine learning systems in a clinical setting might not only streamline data processing but also improve the objective classification of patients at high risk of a complicated ICU stay. This could lead to more proactive patient management.

To fully realize the potential of machine learning and deep learning in this realm, efforts should be made to leverage the capabilities of large international open-source aSAH databases. Such collaborative endeavours would provide a stable, expert-independent rating system and could serve as a benchmark for refining predictive algorithms in the future.

## Electronic supplementary material

Below is the link to the electronic supplementary material.

Supplementary Material 1

## Data Availability

The complete preprocessing procedure can be accessed via the publicly available GitHub repository: https://github.com/agschweingruber/sah. Data Availability: The datasets generated and analysed during this study will be made available to researchers upon reasonable request, in accordance with our data sharing policy and subject to ethical and data protection regulations.

## Abbreviations

AUC: Area Under the Curve
aSAH: aneurysmal Subarachnoid Haemorrhage
BGA: Blood Gas Analysis
CI: Confidence Interval
CSF: Cerebrospinal Fluid
CRP: C-reactive protein
CTP: Computed Tomography Perfusion
DCI: Delayed Cerebral Ischemia
DSA: Digital Subtraction Angiography
EVD: External ventricular drainage
FPR: False Positive Rate
GCS: Glasgow Coma Scale
ICP: Intracranial Pressure
ICU: Intensive Care Unit
IG: Integrated Gradients
LD: Lumbar drain
LSTM: Long Short-Term Memory
ML: Machine Learning
MLP: Multi-Layer Perceptron
NCHCT: Non-contrast Head CT
pCO2: Carbon Dioxide Partial Pressure
PR: Precision Recall
ReLu: Rectified Linear unit
RNN: Recurrent Neural Network
ROC: Receiver Operating Characteristics
SAH: Subarachnoid Haemorrhage
TPR: True Positive Rate
VP: ventriculoperitoneal
XGBoost: Extra Gradient Boosted Machine

## Declarations

We confirm that our manuscript adheres to all the specified instructions and guidelines outlined for authors. The manuscript has been prepared in accordance with the journal’s formatting and stylistic requirements.

We hereby confirm and attest that this manuscript has not been previously published in any form or medium and is not concurrently under consideration by another scientific journal.

We hereby confirm that all individuals listed as authors have significantly contributed to the development of this manuscript and satisfy the authorship criteria. Further, we affirm that the final version of the manuscript has been reviewed and approved by all authors.

### Use of reporting Checklist

For the present study, the RECORD (REporting of studies Conducted using Observational Routinely collected Data) checklist was rigorously followed to ensure robust reporting standards. The completed RECORD checklist will be uploaded concomitantly with the manuscript submission.
