# Supplementary material for: Early prediction of ventricular peritoneal shunt dependency in aneurysmal subarachnoid haemorrhage patients by recurrent neural network-based machine learning using routine intensive care unit data
Source: J Clin Monit Comput. 2024 Mar 21;38(5):1175–86. doi: 10.1007/s10877-024-01151-4 (PMC11427477; doi:10.1007/s10877-024-01151-4)
Supplement: Supplementary file 1 — Supplementary Material 1 [file 10877_2024_1151_MOESM1_ESM.docx]

**Supplementary Information**

**Early prediction of ventricular peritoneal shunt dependency in aneurysmal subarachnoid haemorrhage with recurrent neural networks-based machine learning**

[Supplementary Tables 2](#_Toc138072350)

[Supplementary Table 1 – Modell Performance with deceased patients 2](#_Toc138072351)

[Supplementary Table 2 – All included Feature 3](#_Toc138072352)

[Supplementary Table 3 – Model Performance in survived aSAH patients treated on ICU to predict VP-shunt dependency in a temporal train-test split. 6](#_Toc138072353)

[Supplementary Figures 7](#_Toc138072354)

[Supplementary Figure 1 - Prediction of VP-shunt Dependency: Model Performance Over the First Two Weeks including all patients. 7](#_Toc138072355)

[Supplementary Figure 2 – Confusion Matrix for the best performing RNN on day 4 8](#_Toc138072356)

# Supplementary Tables

## Supplementary Table 1 – Modell Performance with deceased patients

|  | Day | Specificity | Sensitivity | Accuracy | AUROC | F1 | Average Precision |
| --- | --- | --- | --- | --- | --- | --- | --- |
|  |  |  |  |  |  |  |  |
| RNN | 1 | 0.67 (0.63-0.7) | 0.75 (0.71-0.79) | 0.68 (0.65-0.7) | 0.71 (0.69-0.74) | 0.35 (0.34-0.36) | 0.28 (0.25-0.31) |
|  | 4 | 0.68 (0.65-0.7) | 0.82 (0.77-0.86) | 0.69 (0.67-0.72) | 0.76 (0.74-0.78) | 0.37 (0.35-0.38) | 0.33 (0.3-0.36) |
|  | 7 | 0.72 (0.69-0.74) | 0.8 (0.75-0.84) | 0.73 (0.71-0.75) | 0.77 (0.75-0.79) | 0.37 (0.36-0.39) | 0.35 (0.32-0.38) |
| XGBoost | 1 | 0.59 (0.51-0.67) | 0.64 (0.53-0.75) | 0.59 (0.53-0.66) | 0.6 (0.52-0.69) | 0.3 (0.29-0.31) | 0.22 (0.19-0.25) |
|  | 4 | 0.66 (0.62-0.69) | 0.76 (0.72-0.79) | 0.67 (0.64-0.7) | 0.71 (0.69-0.74) | 0.35 (0.34-0.36) | 0.3 (0.27-0.34) |
|  | 7 | 0.65 (0.53-0.78) | 0.72 (0.64-0.8) | 0.66 (0.56-0.77) | 0.7 (0.63-0.76) | 0.34 (0.34-0.35) | 0.26 (0.23-0.29) |
| LogReg | 1 | 0.62 (0.58-0.66) | 0.66 (0.61-0.71) | 0.63 (0.59-0.66) | 0.64 (0.6-0.67) | 0.31 (0.3-0.33) | 0.25 (0.22-0.27) |
|  | 4 | 0.68 (0.65-0.71) | 0.76 (0.72-0.81) | 0.69 (0.67-0.71) | 0.74 (0.71-0.76) | 0.36 (0.35-0.36) | 0.32 (0.3-0.34) |
|  | 7 | 0.7 (0.67-0.72) | 0.78 (0.76-0.81) | 0.71 (0.68-0.73) | 0.75 (0.73-0.76) | 0.37 (0.36-0.37) | 0.34 (0.31-0.36) |
| Fisher | 1 | 0.38 (0.35-0.41) | 0.83 (0.77-0.89) | 0.44 (0.41-0.46) | 0.62 (0.59-0.64) | 0.25 (0.24-0.27) | 0.17 (0.16-0.18) |
| Graeb | 1 | 0.72 (0.67-0.77) | 0.65 (0.56-0.73) | 0.71 (0.67-0.75) | 0.68 (0.64-0.73) | 0.32 (0.3-0.35) | 0.25 (0.22-0.28) |
| Hunt&Hess | 1 | 0.61 (0.59-0.64) | 0.65 (0.59-0.7) | 0.62 (0.6-0.64) | 0.63 (0.61-0.65) | 0.31 (0.3-0.32) | 0.17 (0.16-0.18) |

AUC-ROC = area under the receiver operating characteristic curve; LogReg = logistic regression; RNN = recurrent neural network; XGBoost = Extreme Gradient Boosting; Depicted values are the mean and the confidence intervals calculated based on the prediction of six independent testing folds.

## Supplementary Table 2 – All included Feature

| **Feature Label** | **English Translation** |
| --- | --- |
| BGA_Ca | BGA (Blood Gas Analysis) Calcium |
| BGA_FiO2_BGA | BGA (Blood Gas Analysis) Fraction of inspired oxygen |
| BGA_Glu | BGA (Blood Gas Analysis) Glucose |
| BGA_Hb_BGA | BGA (Blood Gas Analysis) Haemoglobin |
| BGA_HCO3 | BGA (Blood Gas Analysis) Bicarbonate |
| BGA_K | BGA (Blood Gas Analysis) Potassium |
| BGA_Lac | BGA (Blood Gas Analysis) Lactate |
| BGA_Na | BGA (Blood Gas Analysis) Sodium |
| BGA_PaO2/FiO2 | BGA (Blood Gas Analysis) Ratio of arterial oxygen partial pressure to fractional inspired oxygen |
| BGA_PCO2 | BGA (Blood Gas Analysis) Partial pressure of carbon dioxide |
| BGA_pH | BGA (Blood Gas Analysis) pH |
| BGA_PO2 | BGA (Blood Gas Analysis) Partial pressure of oxygen |
| BGA_SBE | BGA (Blood Gas Analysis) Standard base excess |
| BGA_sO2 | BGA (Blood Gas Analysis) Oxygen saturation |
| BGA_Cl | BGA (Blood Gas Analysis) Chloride |
| BGA_Bili | BGA (Blood Gas Analysis) Bilirubin |
| BGA_FCOHb | BGA (Blood Gas Analysis) Carboxyhemoglobin |
| BGA_Temp_BGA | BGA (Blood Gas Analysis) Temperature |
| Labor_Albumin | Laboratory Albumin |
| Labor_Alk._Phospatase | Laboratory Alkaline Phosphatase |
| Labor_ALT_(GPT) | Laboratory Alanine Transaminase (Glutamic Pyruvic Transaminase) |
| Labor_aPTT | Laboratory Activated Partial Thromboplastin Time |
| Labor_AST_(GOT) | Laboratory Aspartate Transaminase (Glutamic Oxaloacetic Transaminase) |
| Labor_Bilirubin_gesamt | Laboratory Total Bilirubin |
| Labor_CK | Laboratory Creatine Kinase |
| Labor_CRP | Laboratory C-Reactive Protein |
| Labor_Erythrocyten | Laboratory Erythrocytes |
| Labor_EVB | Laboratory Erythrocyte Distribution Width |
| Labor_Fibrinogen | Laboratory Fibrinogen |
| Labor_fT3 | Laboratory Free T3 |
| Labor_fT4 | Laboratory Free T4 |
| Labor_GFR | Laboratory Glomerular Filtration Rate |
| Labor_GGT | Laboratory Gamma Glutamyl Transferase |
| Labor_Harnstoff' | Laboratory Urea |
| Labor_Hb | Laboratory Haemoglobin |
| Labor_Hk | Laboratory Haematocrit |
| Labor_INR | Laboratory International Normalized Ratio |
| Labor_Kreatinin | Laboratory Creatinine |
| Labor_LDH | Laboratory Lactate Dehydrogenase |
| Labor_Leukocyten | Laboratory Leukocytes |
| Labor_Lipase | Laboratory Lipase |
| Labor_Magnesium | Laboratory Magnesium |
| Labor_MCH | Laboratory Mean Corpuscular Haemoglobin |
| Labor_MCHC | Laboratory Mean Corpuscular Haemoglobin Concentration |
| Labor_MCV | Laboratory Mean Corpuscular Volume |
| Labor_pankreasspez._Amylase | Laboratory Pancreatic Specific Amylase |
| Labor_Phosphat_anorg. | Laboratory Inorganic Phosphate |
| Labor_Quick | Laboratory Quick's test (prothrombin time) |
| Labor_Thrombocyten | Laboratory Platelets |
| Labor_Triglyceride | Laboratory Triglycerides |
| Labor_TSH_basal | Laboratory Basal Thyroid Stimulating Hormone |
| Labor_TZ | Laboratory Thrombin Time |
| Labor_Cholesterin | Laboratory Cholesterol |
| Labor_PCT | Laboratory Procalcitonin |
| Labor_Vancomycin_vor_Gabe | Laboratory Vancomycin Before Administration |
| Labor_Basophile | Laboratory Basophils |
| Labor_Lymphocyten | Laboratory Lymphocytes |
| Labor_Eiweiß_gesamt | Laboratory Total Protein |
| Labor_CK_MB_(U/l) | Laboratory Creatine Kinase MB |
| Labor_Troponin_T | Laboratory Troponin T |
| Labor_Lactat | Laboratory Lactate |
| Labor_Liquor:_Gesamteiweiß | CSF Total Protein |
| Labor_Liquor:_Glucose | CSF Glucose |
| Labor_Liquor:_Granulozyten | CSF Granulocytes |
| Labor_Liquor:_IL6 | CSF Interleukin 6 |
| Labor_Liquor:_Lymphozyten | CSF Lymphocytes |
| Labor_Liquor:_Monozyten | CSF Monocytes |
| Labor_Liquor:_Zellzahl/Leukozyten | CSF Cell Count/Leukocytes |
| Labor_NSE | Laboratory Neuron-Specific Enolase |
| Labor_TSH | Laboratory Thyroid Stimulating Hormone |
| Labor_Harnstoff_N | Laboratory Urea Nitrogen |
| Labor_Ethanol_enzym._Blut | Laboratory Ethanol Enzyme Blood |
| Labor_Pat._Temperatur | Laboratory Patient Temperature |
| Vital_AF | Vital Breaths per Minute |
| Vital_AMV_total' | Vital Total Minute Volume |
| Vital_ASB | Vital Assisted Spontanous Breathing |
| Vital_Ausfuhr | Vital Excretion |
| Vital_Ausfuhr_Magensonde' | Vital Excretion Gastric Tube |
| Vital_AZV | Vital Minute Ventilation |
| Vital_BDK | Vital Urine Catheter |
| Vital_Beatmungsmodus | Vital Ventilation Mode |
| Vital_Bilanz | Vital Balance |
| Vital_Bilanz_mit_Blut+Liquor | Vital Balance with Blood + CSF |
| Vital_Bilanz_seit_Aufnahme | Vital Balance Since Admission |
| Vital_Bilanz_um_6:00_Uhr | Vital Balance at 6:00 AM |
| Vital_BIS | Vital Bispectral Index |
| Vital_diast | Vital Diastolic Blood Pressure |
| Vital_Einfuhr | Vital Intake |
| Vital_EVD | Vital External Ventricular Drainage |
| Vital_EVD_drain | Vital EVD Drainage |
| Vital_EVD_niveau | Vital EVD Level |
| Vital_FiO2 | Vital Fraction of Inspired Oxygen |
| Vital_Flow_Trigger | Vital Flow Trigger |
| Vital_Freq_gesamt | Vital Total Frequency |
| Vital_Freq_spontan | Vital Spontaneous Frequency |
| Vital_GCS_auge | Vital Glasgow Coma Scale Eye Response |
| Vital_GCS_motor | Vital Glasgow Coma Scale Motor Response |
| Vital_GCS_total | Vital Total Glasgow Coma Scale |
| Vital_GCS_verbal | Vital Glasgow Coma Scale Verbal Response |
| Vital_HF | Vital Heart Rate |
| Vital_I_E | Vital Inspiratory/Expiratory Ratio |
| Vital_Insp_Druck | Vital Inspiratory Pressure |
| Vital_Lagerung | Vital Positioning |
| Vital_Lichtreaktion_li/re | Vital Light Reaction Left/Right |
| Vital_mittl | Vital Mean Blood Pressure |
| Vital_oraler_Tubus | Vital Oral Tube |
| Vital_PEEP | Vital Positive End-Expiratory Pressure |
| Vital_Pmean | Vital Mean Airway Pressure |
| Vital_Ppeak | Vital Peak Airway Pressure |
| Vital_Pulsation | Vital Pulsation |
| Vital_Pupille_li | Vital Pupil Left |
| Vital_Pupille_re | Vital Pupil Right |
| Vital_Residualvolumen_MS_Ausfuhr | Vital Residual Volume Gastric Tube Excretion |
| Vital_Residualvolumen_MS_Einfuhr | Vital Residual Volume Gastric Tube Intake |
| Vital_SpO2 | Vital Peripheral Oxygen Saturation |
| Vital_syst | Vital Systolic Blood Pressure |
| Vital_t_insp | Vital Inspiratory Time |
| Vital_Temp | Vital Temperature |
| Vital_Urinproduktion | Vital Urine Production |
| Vital_ZVD | Vital Central Venous Pressure |
| Vital_CPP | Vital Cerebral Perfusion Pressure |
| Vital_ICP | Vital Intracranial Pressure |
| Vital_Liquor_Ausfuhr_gesamt | Vital Total CSF Excretion |
| Vital_Liquor_Drainage | Vital CSF Drainage |
| Vital_Mobilisation | Vital Mobilization |
| Vital_diast_NBD | Vital NBD Diastolic Blood Pressure |
| Vital_mittl_NBD | Vital NBD Mean Blood Pressure |
| Vital_Resp | Vital Respiration |
| Vital_Spontanurin | Vital Spontaneous Urination |
| Vital_syst_NBD | Vital NBD Systolic Blood Pressure |
| Vital_ATC | Vital Automated Tubus Compensation |
| Vital_Tagesbilanz_Ziel | Vital Daily Balance Target |
| Vital_Kcal_Zufuhr_in_24h | Vital Kcal Intake in 24 hours |
| Vital_RASS | Vital Richmond Agitation-Sedation Scale |
| Vital_arterielle_BGA | Vital Arterial Blood Gas Analysis |
| Vital_Exsp_CO2 | Vital End Tidal CO2 |
| Vital_Behavioral_Pain_Scale | Vital Behavioral Pain Scale |
| Vital_Delir_Score | Vital Delirium Score |
| Vital_Dynamische_Compliance | Vital Dynamic Compliance |
| Vital_DRG_Einfuhr_Infusionen | Vital DRG Infusions Intake |
| Vital_DRG_Ausfuhr_Urin | Vital DRG Urine Output |
| Vital_DRG_Einfuhr_Perfusor_Kurzinfusion | Vital DRG Perfusor Short Infusion Intake |
| Vital_RASS_Ziel | Vital RASS Target |
| Vital_Numeric_Rating_Scale | Vital Numeric Rating Scale |
| Vital_Puls | Vital Pulse |
| Größe | Height |
| Gewicht | Weight |
| Alter | Age |
| Geschlecht | Gender |
| HuntandHess | Hunt and Hess Score |
| Fisher | Fisher Score |
| Graeb | Graeb Score |

## Supplementary Table 3 – Model Performance in survived aSAH patients treated on ICU to predict VP-shunt dependency in a temporal train-test split.

|  | Day | Specificity | Sensitivity | Accuracy | AUROC | F1 | Average  Precision |
| --- | --- | --- | --- | --- | --- | --- | --- |
|  |  |  |  |  |  |  |  |
| RNN | 1 | 0.71 | 0.83 | 0.73 | 0.80 | 0.3 | 0.46 |
|  | 4 | 0.78 | 0.83 | 0.78 | 0.85 | 0.40 | 0.60 |
|  | 7 | 0.87 | 0.75 | 0.85 | 0.83 | 0.40 | 0.61 |
| XGBoost | 1 | 0.59 | 0.75 | 0.61 | 0.65 | 0.3 | 0.20 |
|  | 4 | 0.68 | 0.92 | 0.72 | 0.80 | 0.39 | 0.39 |
|  | 7 | 0.76 | 0.75 | 0.76 | 0.76 | 0.38 | 0.29 |
| LogReg | 1 | 0.63 | 0.67 | 0.64 | 0.66 | 0.32 | 0.2 |
|  | 4 | 0.67 | 0.92 | 0.7 | 0.78 | 0.39 | 0.38 |
|  | 7 | 0.71 | 0.83 | 0.73 | 0.78 | 0.38 | 0.47 |
| Fisher | 1 | 0.55 | 0.58 | 0.56 | 0.62 | 0.28 | 0.18 |
| Graeb | 1 | 0.92 | 0.25 | 0.83 | 0.59 | 0.20 | 0.21 |
| Hunt&Hess | 1 | 0.78 | 0.505 | 0.74 | 0.63 | 0.30 | 0.19 |

AUC-ROC = area under the receiver operating characteristic curve; LogReg = logistic regression; RNN = recurrent neural network; XGBoost = Extreme Gradient Boosting. Depicted values are the prediction on one temporal train-test split. 401 patients from 2010 to 2016 were designated to the training split, 101 patients from 2016 to 2018 were designated to the validation split and 101 patients from 2018 to 2020 were designated to the test split.

# Supplementary Figures


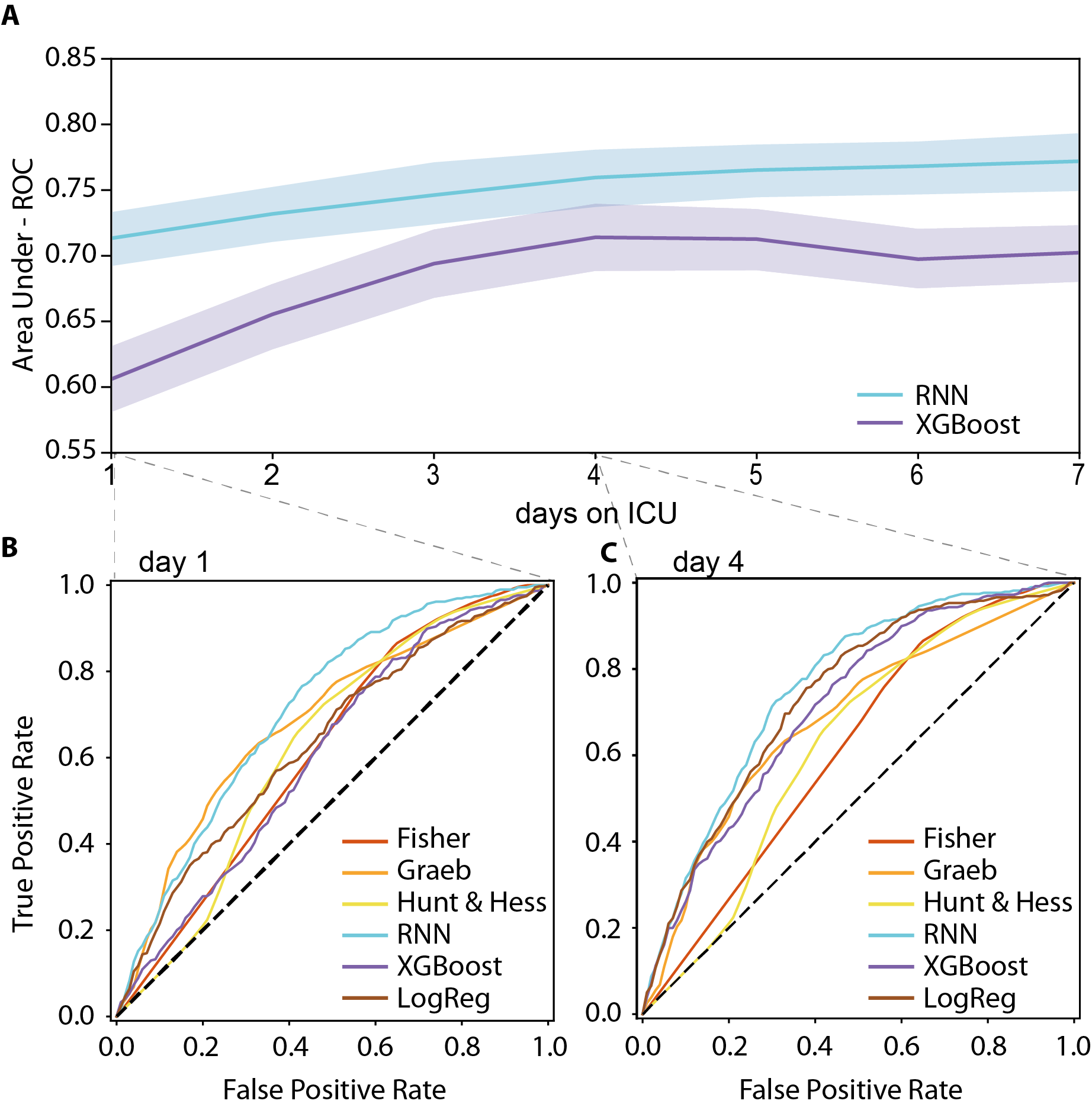


## Supplementary Figure 1 - Prediction of VP-shunt Dependency: Model Performance Over the First Two Weeks including all patients.

The figure represents the performance of two machine learning models, recurrent neural network (RNN) and Extreme Gradient Boosting (XGBoost), in predicting VP-Shunt dependency in aSAH patients admitted to the ICU. The figure is comprised of three subplots (A-C). Subplot A shows the performance of the models over the first 7 days after ICU admission, with the RNN model represented by a blue line and the XGBoost model represented by a purple line. The lighter shaded area represents the Confidence Interval (CI) of the model performance. Subplot B presents the Area Under the Curve (AUC) Receiver Operating Characteristic (ROC) plot for day 1 performance, while Subplot C presents the AUC-ROC plot for day 4 performance. The RNN and XGBoost models are represented by the same colors as in Subplot A, while the aSAH scores are depicted as Fisher score in red, Grab score in orange,Hunt and Hess score in yellow and logistic regression in brown. It is worth noting that the CI is intentionally left out of subplots B and C to improve readability and can be found in Supp. Table 1.


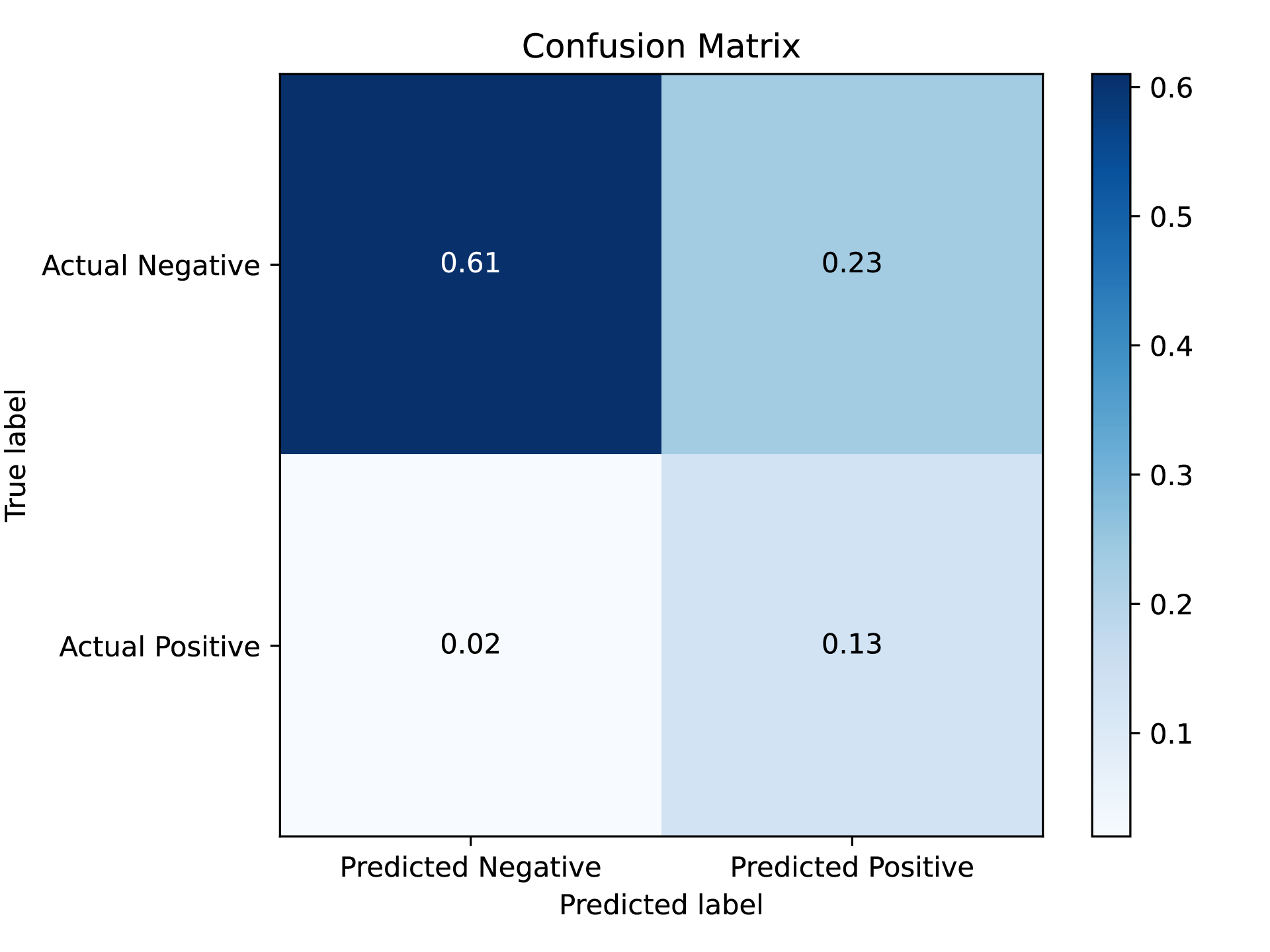


## Supplementary Figure 2 – Confusion Matrix for the best performing RNN on day 4
